# The association of physician assistant/associate demographic and practice characteristics with perceptions of value of certification

**DOI:** 10.1186/s12909-023-04215-2

**Published:** 2023-04-11

**Authors:** Andrzej Kozikowski, Dawn Morton-Rias, Kasey Puckett, Colette Jeffery, Sheila Mauldin, Joshua Goodman

**Affiliations:** National Commission on Certification of Physician Assistants, 12000 Findley Road, Suite 100, Johns Creek, GA 30097 USA

**Keywords:** Physician assistants, Physician associate, PA, Recertification, Value of certification, Perceptions of certification

## Abstract

**Background:**

To determine physician assistant/associate (PA) perceptions of the value of certification and explore how they vary across demographic and practice characteristics.

**Methods:**

We conducted a cross-sectional online survey between March and April 2020 with PAs participating in the longitudinal pilot program for recertification administered by the National Commission on Certification of Physician Assistants (NCCPA). The survey was distributed to 18,147 PAs, of which 10,965 participated (60.4% response rate). In addition to descriptive statistics, chi-square tests were conducted on demographics and specialty to examine if perceptions of value of certification (1 global and 10 items measuring specific domains) were associated with a particular PA profile. A series of fully adjusted multivariate logistic regressions were performed, exploring the relationship between PA characteristics and the value of certification items.

**Results:**

Most PAs strongly agreed/agreed that certification helps with fulfilling licensure requirements (9,578/10,893; 87.9%), helps with updating medical knowledge (9,372/10,897; 86.0%), and provides objective evidence of continued competence (8,875/10,902; 81.4%). The items receiving the lowest percentage of responses for strongly agreeing/agreeing were for certification providing no value (1,925/10,887; 17.7%), helping with professional liability insurance (5,076/10,889; 46.6%), and competing with other providers for clinical positions (5,661/10,905; 51.9%). Age 55 and older and practicing in dermatology and psychiatry were among the strongest predictors of less favorable views. PAs from underrepresented in medicine (URiM) backgrounds had more positive perceptions.

**Conclusions:**

Overall, the findings indicate that PAs value certification; however, perceptions varied by demographics and specialties. PAs who were younger, from URiM backgrounds, and practicing in primary care specialties had among the most favorable perspectives. Continued feedback monitoring is critical in ensuring certification is relevant and meaningful in supporting PAs across demographics and specialties. Measuring PA perceptions of the value of certification is essential to understanding how to support the PA profession's current and future credentialing needs and those who license and hire PAs.

## Background

The National Commission on Certification of Physician Assistants (NCCPA) serves the purpose of ensuring that Board Certified physician assistants/associates (PAs) demonstrate the medical knowledge necessary for certification for entry into the profession and throughout the trajectory of their careers. PAs undergo rigorous education encompassing a general didactic curriculum with clinical rotations and certify and recertify on core medical knowledge for a generalist. As part of the requirements for obtaining a license to practice medicine, PAs are required to complete their studies in an accredited PA program and attain certification by passing the Physician Assistant National Certifying Exam (PANCE). The rapid pace with which medical science develops necessitates that PAs continually acquire emerging knowledge and stay current with evolving standards of care. To maintain certification, PAs are required to accumulate continuing medical education credits every two years and attain a passing score on the Physician Assistant National Recertifying Exam (PANRE) or the Physician Assistant National Recertifying Exam Longitudinal Assessment (PANRE-LA) every ten-years. Both PANCE and PANRE are secure summative examinations. Prior to 2019, the only option for PA recertification was to attain a passing score on the traditional PANRE; this is a secure four-hour exam taken at a testing center and consists of 240 multiple-choice questions. In 2020, NCCPA completed a two-year longitudinal pilot program as an alternative to the PANRE. Although also a summative exam, the pilot program incorporated formative elements to promote ongoing learning. Key components included spaced testing, individualized feedback, and the provision of tailored learning resources contingent on performance. The longitudinal assessment, PANRE-LA is similar to the pilot program and was launched as an official alternative path for recertification in 2023. PANRE-LA is administered online over 12 quarters (25 questions are released per quarter with 5 min per question) but can be completed in 8 quarters if a passing score is reached. PAs can access resources while taking the spaced exam and are provided with feedback to identify knowledge gaps and resources for further learning.

NCCPA seeks to continuously improve its certification program in response to feedback from stakeholders, including the public and PAs, and advances in medical practice, learning science, psychometrics, and technology. To optimally meet the needs of PAs, information regarding their perceptions of overall value toward certification and specific aspects is critical. However, to our knowledge, no previous published study has explored how PAs perceive the value of NCCPA certification.

Evidence exists on how the public views PA certification and medical education. A recent survey revealed that 76% knew that PAs must pass a national certifying exam as one prerequisite for receiving an initial license to practice medicine [1]. It was also observed that most agreed that PAs should continually acquire new medical knowledge (92%), be evaluated at regular intervals (82%), be held to the same standards of care as physicians (79%) and that PAs are well-educated in medicine (78%) [1]. These findings are consistent with how the public values and supports certification of physicians [2].

Substantially more research has been undertaken to investigate how physicians view initial certification and maintenance of certification (MOC) [3–13]. For initial certification, one study demonstrated that among physicians taking the American Board of Emergency Medicine (ABEM) Oral Certification Examination (OCE), 92% indicated that it provided a career benefit, and 80% said preparing for and taking the exam fostered learning [5]. Culley et al. found that 97% of anesthesiologists in their study agreed that certification is valuable for employment opportunities, and 69% believed that certified physicians are more competent than those who are uncertified [10]. The authors also examined anesthesiologists' perceptions of MOC in anesthesiology, finding that the majority perceived Lifelong Learning and Self-Assessment (81%), Professional Standing (76%), Cognitive Examination (61%), and Practice Performance and Improvement (59%) components of MOC as relevant to their practice [10].

A 2021 scoping review based on 125 studies concluded that physicians support MOC in that it encourages lifelong learning; at the same time, the authors noted that physicians expressed dissatisfaction with specific aspects of these programs [13]. One reoccurring concern centered on MOC components and relevance to practice [13]. Further, in a national survey with 988 physicians across different specialties, it was found that 38% agreed that studying for MOC assessments contributes to professional development, and 21% indicated that it improves patient safety [3]. However, fewer (15%) agreed that MOC is worth the time and effort required.

Several studies assessed perceptions of the value of MOC for physicians in specific specialties. Lipner et al. found that 45% to 61% of internal medicine physicians reported partaking in MOC to uphold a professional image, increase knowledge, and sustain or improve patient care quality [11]. Likewise, Marco et al. identified that emergency medicine physician perceptions of the benefits of the Continuous Certification (ConCert) exam comprised reinforced medical knowledge (74%), more employment options (74%), increased knowledge (67%), being more positively regarded by other physicians (57%), being a better clinician (39%), and improved financial outcomes (30%) [7]. Similarly, Peabody and colleagues explored why family physicians seek to continue their American Board of Family Physicians (ABFM) certification [4]. Top reasons identified were that it was required for hospital privileges/credentialing (56%), maintains professional image (55%), was a personal preference (53%), and helps in updating medical knowledge (52%). Slightly fewer stated certification helps monitor or improve patient care quality (42%) and professional advancement (35%). In a study examining rheumatologist perspectives concerning the value of MOC on practice and patient care, it was determined that 64% did not indicate MOC was valuable in terms of improving patient care; nonetheless, 66% confirmed that staying up-to-date with new medical knowledge was a benefit [8].

In a survey assessing Certified Registered Nurse Anesthetist (CRNA) views of the certification credential, 92% responded that certification was valuable to them [14]. Further, 96% indicated it provides personal satisfaction, 85% reported it validates their comprehension of core knowledge needed to practice nurse anesthesia, and 82% said the credential enhanced their expertise.

As seen in the existing literature with physicians practicing in different specialties and CRNAs, the pattern of findings regarding certification perceptions is mixed; however, one of the most noted benefits is that certification helps validate, reinforce or update medical knowledge. Yet, PA perspectives regarding certification and potential benefits remain unknown. Moreover, it was unclear whether perceptions vary by PA demographic attributes and disciplines, which is an important element in evaluating the value of certification. For this reason, we conducted an online survey with PAs to shed light on the following three research questions: 1) Overall, do PAs value certification, and are specific benefits more prevalent than others? 2) Do perceptions of the value of certification vary based on PA demographics and specialties? 3) Which demographic attributes and specialties are significant independent predictors, and what is their relative importance in valuing certification?

## Methods

A cross-sectional online survey study of PAs participating in NCCPA's longitudinal pilot program was conducted from March through April 2020. This study was reviewed and deemed exempt by Sterling IRB (#8310). The pilot launched in January 2019 with 18,529 PAs volunteering to participate, representing approximately 58% of the PAs due for recertification in 2018 and 2019. The participant group was representative of those eligible for recertification based on age groups, gender, race, geographic region, practice area (primary care, surgery, other), years working as a PA, and clinical employment. At the close of the pilot in December 2020, 97.7% (18,099) of PAs had remained in the process for the full two-year pilot test. The large majority of withdrawals were due to PAs not completing their requirements. For the 65 PAs who chose to voluntarily withdraw, the two reasons cited most frequently were (1) retiring and (2) preferring to take the exam all at once instead of spaced over two years. PAs participating in the pilot were provided quarterly surveys over two years to garner feedback regarding the testing experience and were provided a disclaimer indicating that responses would be confidential and reported in aggregate to encourage honest feedback.

During the seventh quarter (March–April 2020), we added questions to explore PA perceptions related to the value of NCCPA certification. We designed the questions to elicit PA perspectives of NCCPA certification generally rather than a specific program (i.e., PANCE, PANRE, or longitudinal pilot). Survey development was informed by prior studies exploring value of certification perceptions [3, 4]. Questions were added or iteratively modified until a final set of 11 closed-ended items (one global value judgment and 10 queried various aspects of valuing certification) were agreed upon by study authors who have specialized training and experience in survey development. The response scale was based on a Likert-type agreement (strongly agree, agree, disagree, strongly disagree, and no opinion). The following are introductory instructions and the final set of items:

"In addition to providing feedback on the Pilot program, we would like to ask you to provide your perspectives on the value you believe that PA certification provides. Indicate your level of agreement with each of the following statements as they relate to your perception of the value of certification."

- Provides no value to me as a PA
- Helps with fulfilling my licensure requirements
- Helps me update my medical knowledge
- Provides objective evidence of my continued level of competence
- Contributes to the acceptance/respect others have of the PA profession
- Provides personal satisfaction
- Helps me monitor or improve the quality of my patient care
- Helps with the credentialing process at my hospital or institution
- Helps me to advance professionally
- Helps me to better compete with other providers such as NPs for clinical positions
- Helps with my professional liability insurance

We also added an open-ended item, "Please provide any additional feedback you would like to share related to the value of NCCPA certification," where PAs could write in their responses. This provided us with the opportunity for richer examinations of perspectives surrounding certification. The value of certification items were programmed in the Qualtrics survey platform and presented in a randomized order to limit order effects or the influence of initial items on responses to subsequent ones [15]. To minimize the survey length, PA demographic and practice characteristics, including age, gender, race/ethnicity, and specialty, were obtained from NCCPA's PA Professional Profile [16, 17] and merged with responses in the survey. We distributed a survey link to 18,147 PAs, of which 10,965 responded. In an effort to increase the response rate, one reminder email was sent before closing the survey (response rate was 60.4%).

We first calculated descriptive statistics for all variables. To conduct inferential analyses, the five-category agreement scales evaluating value of certification were collapsed into two mutually exclusive categories: "strongly agree" and "agree" into "agree," and all others into "not agree." Chi-square tests of independence were conducted on all demographic characteristics and specialty to examine if perceptions of value of certification were associated with a particular PA profile. A series of fully adjusted multivariate logistic regressions were performed, exploring the relationship between PA characteristics and each of the value of certification items. Multicollinearity was assessed using variance inflation factor, showing model fit was not affected. All quantitative analyses were conducted using R version 3.6.3 (R Project for Statistical Computing). Statistical significance was set at *P* < 0.05, and all tests were 2-tailed. Qualitative thematic analyses of responses to the open-ended item were conducted in NVIVO (QSR International) to provide insights into the quantitative findings.

## Results

### Descriptive statistics of PA characteristics and value of certification items

Table 1 presents PA demographic characteristics. The highest proportion (35.5%) were in the 35 to 44 age group (3,892/10,965). The majority were female (7,551/10,965; 68.9%), white (9,007/10,207; 88.2%), and non-Hispanic/Latino (10,256/10,870; 94.4%). With regard to specialties, the highest percentage practiced in primary care (2,518/10,706; 23.5%). Demographics and specialties of participants in the study sample were similar to that of the overall nationally certified PA population in terms of age (30–39 age group; 38.3%), gender (female; 69.7%), race (white; 80.8%), Hispanic/Latino (6.5%) and practicing in primary care (24.4%) [18]. On value of certification (see Tables 2 and 3), most PAs either strongly agreed or agreed that certification helps with fulfilling licensure requirements (9,578/10,893; 87.9%), helps with updating medical knowledge (9,372/10,897; 86.0%), and provides objective evidence of continued level of competencies (8,875/10,902; 81.4%). Items that had the lowest percentage of PAs indicating strongly agreeing or agreeing were for certification providing no value (1,925/10,887; 17.7%), helping with professional liability insurance (5,076/10,889; 46.6%), and helping to compete with other providers for clinical positions (5,661/10,905; 51.9%).

**Table 1:** Physician assistant/associate characteristics

Characteristic	No. (%) (*N* = 10965)
Age	
Less than 35	2725 (24.9)
35–44	3892 (35.5)
45–54	2540 (23.2)
55 +	1808 (16.5)
Gender	
Female	7551 (68.9)
Male	3414 (31.1)
Race	
White	9007 (88.2)
Asian	493 (4.8)
Black/African American	312 (3.1)
Other	395 (3.9)
Ethnicity	
Non-Hispanic/Latino	10256 (94.4)
Hispanic/Latino	614 (5.7)
Specialty	
Primary Care	2518 (23.5)
Surgery–Subspecialties	1885 (17.6)
Emergency Medicine	1219 (11.4)
Internal Medicine–Subspecialties	1039 (9.7)
Dermatology	539 (5.0)
Surgery–General	295 (2.8)
Hospital Medicine	265 (2.5)
Pain Medicine	200 (1.9)
Psychiatry	196 (1.8)
Occupational Medicine	178 (1.7)
Obstetrics and Gynecology	143 (1.3)
Pediatrics-Subspecialties	137 (1.3)
Critical Care Medicine	120 (1.1)
Other	1972 (18.4)

**Table 2:** Physician assistants/associates who agreed or strongly agreed with the following value of certification items and associated characteristics

	Helps with fulfilling my licensure requirements		Helps me update my medical knowledge		Provides objective evidence of my continued level of competence		Contributes to the acceptance/ respect others have of the PA profession		Provides personal satisfaction		Helps me monitor or improve the quality of my patient care	
All No. (%)	9578 (87.9)		9372 (86.0)		8875 (81.4)		8146 (74.7)		7691 (70.6)		7659 (70.3)	
Characteristic	No. (%)	*P* value	No. (%)	*P* value	No. (%)	*P* value	No. (%)	*P* value	No. (%)	*P* value	No. (%)	*P* value
Age		.001		< .001		< .001		< .001		< .001		< .001
Less than 35	2425 (89.5)		2418 (89.2)		2365 (87.2)		2141 (78.9)		2014 (74.3)		2042 (75.2)	
35–44	3400 (87.9)		3338 (86.3)		3212 (83.0)		2884 (74.6)		2660 (68.9)		2732 (70.7)	
45–54	2216 (88.0)		2116 (84.0)		1971 (78.3)		1829 (72.6)		1758 (69.8)		1693 (67.4)	
55 +	1537 (85.5)		1500 (83.4)		1327 (73.7)		1292 (71.7)		1259 (69.9)		1192 (66.2)	
Gender		.150		< .001		< .001		.004		.207		.430
Female	6616 (88.2)		6518 (86.8)		6208 (82.7)		5667 (75.5)		5320 (71.0)		5289 (70.6)	
Male	2962 (87.2)		2854 (84.1)		2667 (78.5)		2479 (73.0)		2371 (69.8)		2370 (69.8)	
Race		.547		.006		.414		.804		.465		.025
White	7879 (88.0)		7705 (86.0)		7326 (81.8)		6745 (75.3)		6339 (70.8)		6287 (70.2)	
Asian	440 (89.8)		438 (89.2)		405 (82.7)		369 (75.3)		355 (72.4)		370 (75.7)	
Black/African American	275 (89.6)		281 (91.8)		263 (85.4)		228 (73.8)		228 (74.5)		229 (74.8)	
Other	343 (87.5)		333 (85.2)		319 (81.4)		289 (73.5)		281 (71.9)		278 (71.3)	
Ethnicity		.194		.064		.172		.141		.038		.015
Non-Hispanic/Latino	8972 (88.0)		8757 (85.9)		8294 (81.3)		7613 (74.7)		7174 (70.4)		7142 (70.1)	
Hispanic/Latino	524 (86.2)		540 (88.7)		511 (83.6)		473 (77.4)		452 (74.5)		455 (74.8)	
Specialty		< .001		.238		< .001		< .001		< .001		< .001
Primary Care	2231 (89.0)		2176 (86.7)		2141 (85.3)		1925 (76.7)		1865 (74.4)		1926 (76.8)	
Surgery–Subspecialties	1676 (89.2)		1614 (86.1)		1464 (77.9)		1345 (71.7)		1280 (68.3)		1201 (64.1)	
Emergency Medicine	1079 (89.5)		1036 (85.8)		1044 (86.6)		956 (79.3)		888 (73.6)		924 (76.7)	
Internal Medicine–Subspecialties	921 (89.0)		904 (87.3)		843 (81.4)		786 (75.8)		719 (69.7)		733 (70.8)	
Dermatology	450 (84.1)		438 (82.2)		388 (72.4)		386 (71.7)		325 (60.7)		315 (58.8)	
Surgery—General	255 (86.7)		257 (87.7)		249 (85.3)		222 (75.8)		208 (71.0)		218 (74.7)	
Hospital Medicine	228 (87.4)		226 (86.6)		226 (85.9)		203 (77.5)		202 (77.1)		202 (77.7)	
Pain Medicine	166 (83.0)		175 (87.5)		164 (82.0)		148 (74.0)		138 (69.0)		145 (72.5)	
Psychiatry	163 (83.6)		160 (82.1)		148 (75.9)		137 (70.3)		120 (61.9)		122 (62.6)	
Occupational Medicine	159 (90.9)		153 (87.4)		134 (76.6)		131 (74.9)		129 (73.7)		134 (77.0)	
Obstetrics and Gynecology	128 (89.5)		125 (87.4)		116 (81.1)		102 (71.3)		93 (65.0)		93 (65.5)	
Pediatrics-Subspecialties	125 (91.9)		122 (89.7)		109 (80.1)		103 (75.7)		102 (75.0)		89 (65.9)	
Critical Care Medicine	104 (87.4)		102 (85.0)		95 (79.2)		87 (72.5)		82 (68.3)		86 (71.7)	
Other	1670 (85.5)		1664 (85.0)		1540 (78.7)		1437 (73.5)		1362 (69.8)		1283 (65.5)	

Results are shown for chi-square tests

**Table 3:** Physician assistants/associates who agreed or strongly agreed with the following value of certification items and associated characteristics

	Helps with the credentialing process at my hospital or institution		Helps me to advance professionally		Helps me to better compete with other providers such as NPs for clinical positions		Helps with my professional liability insurance		Provides no value to me as a PA	
All No. (%)	7547 (69.2)		6326 (58.1)		5661 (51.9)		5076 (46.6)		1925 (17.7)	
Characteristic	No. (%)	*P* value	No. (%)	*P* value	No. (%)	*P* value	No. (%)	*P* value	No. (%)	*P* value
Age		.035		< .001		< .001		< .001		< .001
Less than 35	1898 (70.0)		1833 (67.6)		1585 (58.4)		1379 (50.9)		368 (13.6)	
35–44	2619 (67.7)		2206 (57.1)		2022 (52.2)		1836 (47.5)		665 (17.2)	
45–54	1783 (70.9)		1321 (52.5)		1237 (49.2)		1113 (44.2)		497 (19.8)	
55 +	1247 (69.3)		966 (53.6)		817 (45.3)		748 (41.6)		395 (22.0)	
Gender		< .001		.312		.820		.011		< .001
Female	5096 (68.0)		4380 (58.4)		3902 (52.0)		3555 (47.4)		1182 (15.8)	
Male	2451 (72.2)		1946 (57.4)		1759 (51.7)		1521 (44.8)		743 (21.9)	
Race		.423		< .001		< .001		.002		.004
White	6192 (69.1)		5164 (57.7)		4628 (51.7)		4163 (46.5)		1514 (16.9)	
Asian	342 (69.8)		339 (69.5)		298 (60.8)		267 (54.5)		113 (23.1)	
Black/African American	224 (73.4)		200 (64.9)		179 (58.3)		134 (43.5)		60 (19.5)	
Other	267 (68.3)		230 (58.5)		206 (52.4)		197 (50.3)		65 (16.6)	
Ethnicity		.669		.172		.551		.136		.505
Non-Hispanic/Latino	7052 (69.2)		5902 (57.9)		5290 (51.9)		4726 (46.4)		1809 (17.8)	
Hispanic/Latino	427 (70.1)		371 (60.8)		325 (53.2)		302 (49.6)		101 (16.6)	
Specialty		< .001		< .001		< .001		< .001		.002
Primary Care	1684 (67.2)		1520 (60.6)		1336 (53.2)		1239 (49.4)		391 (15.6)	
Surgery–Subspecialties	1399 (74.6)		1021 (54.3)		938 (50.0)		850 (45.4)		380 (20.3)	
Emergency Medicine	923 (76.5)		767 (63.7)		708 (58.6)		640 (53.2)		206 (17.1)	
Internal Medicine–Subspecialties	752 (72.6)		616 (59.4)		542 (52.2)		473 (45.8)		179 (17.3)	
Dermatology	298 (55.7)		281 (52.6)		264 (49.5)		262 (48.8)		116 (21.6)	
Surgery—General	221 (75.4)		167 (57.0)		150 (51.2)		121 (41.4)		47 (16.0)	
Hospital Medicine	196 (75.1)		177 (67.3)		140 (53.2)		120 (45.6)		50 (19.0)	
Pain Medicine	128 (64.0)		110 (55.6)		101 (50.5)		91 (45.5)		38 (19.1)	
Psychiatry	123 (63.1)		97 (49.7)		95 (48.7)		94 (48.2)		44 (22.6)	
Occupational Medicine	116 (66.3)		103 (59.2)		86 (49.1)		66 (37.7)		33 (19.0)	
Obstetrics and Gynecology	96 (67.6)		81 (56.6)		66 (46.2)		73 (51.0)		28 (19.6)	
Pediatrics-Subspecialties	103 (75.7)		76 (55.9)		72 (52.9)		59 (43.4)		16 (11.8)	
Critical Care Medicine	77 (64.2)		78 (65.5)		70 (58.8)		54 (45.0)		25 (20.8)	
Other	1260 (64.5)		1081 (55.3)		970 (49.6)		822 (42.0)		321 (16.4)	

Results are shown for chi-square tests

### Bivariate associations between PA characteristics and value of certification items

We detected significant differences by age groups to all value of certification items whereby younger PAs consistently had more favorable perceptions (all *P* < 0.05; Tables 2 and 3). When parsing the data by gender, we found that female PAs had significantly more positive perceptions for 5 of the 11 items when compared to males. However, males (2,451/7,547; 72.2%) were more likely than females (5,096/7,547; 68.0%) to indicate that certification helps with the credentialing process (*P* < 0.001). Regarding associations with race, we found that African American PAs were more likely than the other race groups to agree that certification helps update medical knowledge (*P* = 0.006). Significantly higher proportions of Asian PAs agreed that certification helps to monitor or improve quality of patient care, enables advancing professionally, aids in competing with other providers for clinical positions, and assists with professional liability insurance. However, a finding that disrupts this trend was that Asian participants were also more likely to indicate that certification provides no value. Hispanic/Latino PAs had a higher likelihood of agreeing that certification provides personal satisfaction (452/7,626; 74.5% vs. 7,174/7,626; 70.4%; *P* = 0.038) and that it helps to monitor or improve quality of patient care (455/7,597; 74.8% vs. 7,142/7,597; 70.1%; *P* = 0.015).

When examining differences in the value of certification by the specialties in which PAs practice, we found all to be statistically significant (all *P* < 0.05) except for agreeing that certification helps update medical knowledge. PAs practicing in pediatric subspecialties had the highest proportion agreeing that certification helps fulfill licensure requirements. Those in the emergency medicine discipline had the highest percentage stating that certification provides objective evidence of continued competence, contributes to the acceptance/respect others have of the PA profession, helps with the credentialing process in the hospital/institution, and aids with professional liability insurance. Regarding certification providing personal satisfaction, helping to monitor or improve quality of patient care, and supporting professional advancement, PAs working in hospital medicine had the highest proportion of agreement. PAs in critical care medicine had the highest percentage indicating that certification enables them to better compete with other providers for positions. PAs in psychiatry had the highest proportion reporting that certification provides no value.

### Multivariate correlates of increased and decreased odds of agreeing with value of certification items

Tables 4 and 5 depict results of the fully adjusted associations of PA agreement to valuing certification. The relationship between age and value of certification perceptions persisted even after controlling for all covariates. For most items, there was a significant inverse association between age and agreement, such that with increasing age, PAs had progressively lower odds of agreeing. Further, with increasing age, PAs had progressively higher odds of agreeing that certification provides no value. Age 55 and older compared to less than 30 was among the strongest predictors of decreased odds of agreeing that certification helps with updating medical knowledge (adjusted odds ratio [aOR], 0.64; 95% confidence interval [CI] 0.53, 0.77), contributes to the acceptance/respect others have of the PA profession (aOR, 0.68; 95% CI 0.58, 0.79), enables to advance professionally (aOR, 0.55; 95% CI 0.48, 0.63) and helps to better compete with other providers for clinical positions (aOR, 0.61; 95% CI 0.53, 0.70). Moreover, age 55 and older was the strongest predictor of agreeing that certification provides no value (aOR, 1.71; 95% CI 1.44, 2.04).

**Table 4:** Multivariate correlates of increased/decreased odds of agreeing with the following value of certification items

	Helps with fulfilling my licensure requirements		Helps me update my medical knowledge		Provides objective evidence of my continued level of competence		Contributes to the acceptance/respect others have of the PA profession		Provides personal satisfaction		Helps me monitor or improve the quality of my patient care	
Characteristic	aOR	95% CI	aOR	95% CI	aOR	95% CI	aOR	95% CI	aOR	95% CI	aOR	95% CI
Age (less than 35 ref.)												
35–44	0.90	(0.77, 1.06)	0.77*	(0.66, 0.90)	0.74*	(0.63, 0.85)	0.81*	(0.71, 0.91)	0.78*	(0.70, 0.88)	0.79*	(0.70, 0.89)
45–54	0.88	(0.73, 1.06)	0.65*	(0.54, 0.77)	0.55*	(0.47, 0.64)	0.75*	(0.65, 0.86)	0.80*	(0.70, 0.91)	0.67*	(0.59, 0.76)
55 +	0.71*	(0.58, 0.86)	0.64*	(0.53, 0.77)	0.40*	(0.34, 0.48)	0.68*	(0.58, 0.79)	0.82*	(0.71, 0.95)	0.60*	(0.52, 0.70)
Gender (female ref.)												
Male	0.92	(0.8, 1.05)	0.88	(0.78, 1.00)	0.86*	(0.76, 0.97)	0.92	(0.83, 1.02)	0.95	(0.86, 1.05)	1.07	(0.96, 1.18)
Race (White ref.)												
Asian	1.14	(0.85, 1.56)	1.36*	(1.02, 1.86)	0.99	(0.78, 1.27)	1.00	(0.81, 1.25)	1.11	(0.91, 1.38)	1.35*	(1.09, 1.68)
Black/African American	1.17	(0.81, 1.74)	2.02*	(1.34, 3.19)	1.32	(0.96, 1.86)	0.9	(0.69, 1.17)	1.22	(0.94, 1.61)	1.32*	(1.01, 1.74)
Other	1.08	(0.78, 1.53)	0.84	(0.61, 1.16)	0.97	(0.73, 1.31)	0.86	(0.67, 1.11)	0.95	(0.75, 1.23)	0.96	(0.75, 1.23)
Ethnicity (Non-Hispanic/Latino ref.)												
Hispanic/Latino	0.75*	(0.58, 1.00)	1.46*	(1.08, 2.00)	1.15	(0.89, 1.51)	1.19	(0.94, 1.50)	1.28*	(1.02, 1.60)	1.27*	(1.01, 1.59)
Specialty (Primary Care ref.)												
Surgery–Subspecialties	1.03	(0.84, 1.26)	0.94	(0.78, 1.13)	0.57*	(0.48, 0.67)	0.76*	(0.65, 0.87)	0.74*	(0.65, 0.85)	0.51*	(0.44, 0.58)
Emergency Medicine	1.05	(0.83, 1.33)	0.91	(0.74, 1.12)	1.07	(0.87, 1.33)	1.18	(0.99, 1.42)	0.96	(0.81, 1.13)	1.01	(0.85, 1.20)
Internal Medicine–Subspecialties	1.01	(0.80, 1.30)	1.03	(0.82, 1.30)	0.73*	(0.60, 0.90)	0.96	(0.80, 1.15)	0.79*	(0.67, 0.94)	0.73*	(0.61, 0.86)
Dermatology	0.63*	(0.48, 0.83)	0.66*	(0.51, 0.86)	0.38*	(0.30, 0.48)	0.74*	(0.60, 0.93)	0.53*	(0.43, 0.64)	0.40*	(0.32, 0.49)
Surgery—General	0.75	(0.52, 1.10)	0.97	(0.68, 1.44)	0.91	(0.64, 1.32)	0.93	(0.69, 1.26)	0.79	(0.60, 1.05)	0.83	(0.62, 1.12)
Hospital Medicine	0.83	(0.56, 1.26)	0.94	(0.64, 1.42)	0.94	(0.65, 1.41)	1.03	(0.75, 1.43)	1.18	(0.86, 1.63)	1.00	(0.73, 1.39)
Pain Medicine	0.60*	(0.41, 0.92)	1.03	(0.67, 1.65)	0.76	(0.52, 1.14)	0.85	(0.61, 1.20)	0.82	(0.59, 1.14)	0.77	(0.55, 1.08)
Psychiatry	0.68	(0.45, 1.07)	0.64*	(0.43, 0.97)	0.53*	(0.37, 0.77)	0.77	(0.55, 1.09)	0.53*	(0.39, 0.73)	0.49*	(0.36, 0.68)
Occupational Medicine	1.29	(0.77, 2.32)	1.13	(0.71, 1.88)	0.69	(0.47, 1.04)	0.97	(0.68, 1.43)	1.02	(0.71, 1.48)	1.09	(0.75, 1.62)
Obstetrics and Gynecology	1.04	(0.61, 1.91)	0.93	(0.57, 1.60)	0.64	(0.42, 1.02)	0.70	(0.48, 1.04)	0.61*	(0.42, 0.88)	0.54*	(0.38, 0.79)
Pediatrics-Subspecialties	1.56	(0.83, 3.34)	1.22	(0.70, 2.30)	0.56*	(0.37, 0.90)	0.87	(0.58, 1.35)	1.03	(0.69, 1.58)	0.53*	(0.37, 0.78)
Critical Care Medicine	0.80	(0.46, 1.48)	0.74	(0.44, 1.30)	0.57*	(0.36, 0.96)	0.72	(0.47, 1.12)	0.70	(0.47, 1.07)	0.67	(0.44, 1.03)
Other	0.73*	(0.61, 0.88)	0.88	(0.73, 1.05)	0.62*	(0.52, 0.73)	0.82*	(0.71, 0.95)	0.78*	(0.68, 0.90)	0.57*	(0.50, 0.66)

*Abbreviation*: *ref.* indicates reference, *aOR* adjusted odds ratio, *CI* Confidence interval

^*^P < .05

**Table 5:** Multivariate correlates of increased/decreased odds of agreeing with the following value of certification items

	Helps with the credentialing process at my hospital or institution		Helps me to advance professionally		Helps me to better compete with other providers such as NPs for clinical positions		Helps with my professional liability insurance		Provides no value to me as a PA	
Characteristic	aOR	95% CI	aOR	95% CI	aOR	95% CI	aOR	95% CI	aOR	95% CI
Age (less than 35 ref.)										
35–44	0.91	(0.82, 1.02)	0.64*	(0.57, 0.71)	0.79*	(0.71, 0.87)	0.89*	(0.81, 0.99)	1.27*	(1.10, 1.48)
45–54	1.05	(0.92, 1.19)	0.52*	(0.46, 0.59)	0.70*	(0.62, 0.78)	0.79*	(0.70, 0.88)	1.48*	(1.27, 1.74)
55 +	0.96	(0.83, 1.11)	0.55*	(0.48, 0.63)	0.61*	(0.53, 0.70)	0.71*	(0.62, 0.81)	1.71*	(1.44, 2.04)
Gender (female ref.)										
Male	1.13*	(1.03, 1.25)	1.05	(0.96, 1.16)	1.07	(0.98, 1.17)	0.94	(0.86, 1.03)	1.35*	(1.20, 1.52)
Race (White ref.)										
Asian	1.05	(0.86, 1.29)	1.65*	(1.35, 2.03)	1.43*	(1.19, 1.73)	1.35*	(1.12, 1.63)	1.53*	(1.22, 1.90)
Black/African American	1.24	(0.95, 1.62)	1.41*	(1.11, 1.81)	1.34*	(1.06, 1.71)	0.89	(0.70, 1.12)	1.14	(0.84, 1.52)
Other	0.94	(0.74, 1.20)	1.01	(0.80, 1.27)	1.01	(0.81, 1.27)	1.19	(0.95, 1.49)	0.91	(0.67, 1.23)
Ethnicity (Non-Hispanic/Latino ref.)										
Hispanic/Latino	1.05	(0.85, 1.30)	1.17	(0.96, 1.43)	1.06	(0.88, 1.29)	1.09	(0.90, 1.33)	0.99	(0.76, 1.28)
Specialty (Primary Care ref.)										
Surgery–Subspecialties	1.39*	(1.21, 1.60)	0.73*	(0.64, 0.83)	0.82*	(0.72, 0.93)	0.84*	(0.74, 0.95)	1.35*	(1.14, 1.59)
Emergency Medicine	1.62*	(1.37, 1.91)	1.12	(0.96, 1.30)	1.23*	(1.07, 1.43)	1.18*	(1.02, 1.36)	1.09	(0.90, 1.33)
Internal Medicine–Subspecialties	1.37*	(1.16, 1.62)	0.91	(0.78, 1.07)	0.96	(0.82, 1.11)	0.87	(0.75, 1.01)	1.18	(0.96, 1.44)
Dermatology	0.63*	(0.52, 0.76)	0.68*	(0.56, 0.83)	0.82*	(0.68, 1.00)	0.90	(0.75, 1.10)	1.65*	(1.29, 2.10)
Surgery—General	1.48*	(1.11, 1.99)	0.75*	(0.58, 0.97)	0.89	(0.69, 1.15)	0.67*	(0.52, 0.87)	1.07	(0.74, 1.50)
Hospital Medicine	1.51*	(1.12, 2.07)	1.22	(0.92, 1.63)	0.92	(0.71, 1.21)	0.82	(0.63, 1.07)	1.34	(0.94, 1.87)
Pain Medicine	0.82	(0.61, 1.13)	0.78	(0.58, 1.06)	0.85	(0.63, 1.14)	0.89	(0.66, 1.19)	1.31	(0.88, 1.89)
Psychiatry	0.85	(0.62, 1.18)	0.64*	(0.47, 0.87)	0.84	(0.62, 1.14)	1.00	(0.74, 1.36)	1.48*	(1.00, 2.14)
Occupational Medicine	0.93	(0.67, 1.31)	1.02	(0.73, 1.42)	0.91	(0.66, 1.26)	0.68*	(0.49, 0.94)	1.10	(0.71, 1.64)
Obstetrics and Gynecology	1.03	(0.72, 1.50)	0.80	(0.56, 1.13)	0.72	(0.51, 1.01)	1.02	(0.72, 1.44)	1.48	(0.94, 2.27)
Pediatrics-Subspecialties	1.58*	(1.06, 2.43)	0.79	(0.55, 1.14)	0.97	(0.68, 1.39)	0.80	(0.56, 1.14)	0.82	(0.45, 1.38)
Critical Care Medicine	0.86	(0.58, 1.29)	1.05	(0.70, 1.59)	1.18	(0.80, 1.76)	0.80	(0.54, 1.17)	1.55	(0.94, 2.46)
Other	0.88	(0.77, 1.00)	0.78*	(0.68, 0.88)	0.85*	(0.75, 0.96)	0.73*	(0.64, 0.83)	1.04	(0.88, 1.23)

*Abbreviation*: *ref.* indicates reference, *aOR* adjusted odds ratio, *CI* Confidence interval

^*^P < .05

Among the strongest predictors of increased odds of agreeing to many of the items were African American and Asian race and Hispanic/Latino ethnicity. For example, African American PAs compared to white had over two-fold higher odds (95% CI 1.34, 3.19) of agreeing that certification helps update medical knowledge. Likewise, Hispanic/Latino PAs had 1.46 higher odds (95% CI 1.08, 2.00), and Asian PAs had 36% higher odds (95% CI 1.02, 1.86). Being Hispanic/Latino was the strongest predictor of agreeing that certification provides personal satisfaction (aOR, 1.28; 95% CI 1.02, 1.60). Compared to white, Asian, and African American PAs had higher odds of believing that certification helps monitor or improve patient care quality, supports professional advancement, and enables to better compete with other providers for clinical positions.

In terms of practice disciplines, we found that PAs working in two specialties—dermatology and psychiatry—had decreased odds of agreeing with many of the items when compared to PAs in primary care. PAs working in psychiatry had 36% lower odds of agreeing that certification helps update medical knowledge (95% CI 0.43, 0.97) and 47% lower odds of indicating that it provides personal satisfaction (95% CI 0.37, 0.77). Likewise, PAs in the dermatology specialty also had 47% lower odds of reporting that certification provides personal satisfaction (95% CI 0.43, 0.64). Moreover, PAs in dermatology had the strongest decreased odds of agreeing that certification provides objective evidence of continued competencies (aOR, 0.38; 95% CI 0.30, 0.48), helps to monitor or improve the quality of patient care (aOR, 0.40; 95% CI 0.32, 0.49), and helps with credentialing (aOR, 0.63; 95% CI 0.52, 0.76). PAs in dermatology had 65% higher odds of indicating that certification provides no value (95% CI 1.29, 2.10), while those in psychiatry had 48% higher odds (95% CI 1.00, 2.14).

### Qualitative findings from the open-ended question

Of the 10,965 PA survey participants, 923 (8.4%) offered a response. Overall, there were many positive comments toward certification; however, there were also many areas where PAs shared a negative perception. Most felt that initial certification (PANCE) is vital to the profession, but many had mixed views regarding recertification (PANRE). PAs often noted that traditional PANRE is a high-stakes exam causing stress and anxiety and is more difficult to pass with increasing age and specialization. Many participants provided negative remarks regarding the relevance of core medical knowledge on the exam to their practice (particularly in specialty and sub-specialty areas such as psychiatry and dermatology). Conversely, others valued the general content of the exam in that it can enable PAs to change specialties more easily. Many mentioned that NPs do not have to recertify but only maintain CME and suggested that the same should be required of PAs. However, most said the pilot is a step in the right direction and a much better experience than the traditional PANRE. The most prominent benefits of the pilot included that it is less stressful and more convenient, provides feedback and resources that enhance learning, and enables PAs to keep up to date on medical knowledge.

Of 923 comments, 121 (13.1%) were from African American, American Indian/Alaska Native, Asian, Hispanic/Latino, and Native Hawaiian/Pacific Islander PAs. In terms of valuing certification, comments reflected that it provides opportunities, adds legitimacy, respect, and pride; keeps a standard and ensures a certain level of uniform core medical knowledge; and helps to stay competitive, up to date on medical knowledge, and flexible to change specialties.

## Discussion

To the best of the authors' knowledge, this study is the first to gather data on PA perceptions of the value of certification. Among the most important findings that emerged from the analysis is that even among subgroups of PAs who had less favorable views, most do value NCCPA certification. The top benefits reported were that certification helps fulfill licensure requirements, updates medical knowledge, and provides objective evidence of continued competencies. The finding that the highest proportion of PAs indicated certification helps meet licensure requirements is not surprising, as passing PANCE is a prerequisite for initial PA licensure with all state licensing boards. The observation that high proportions of PAs indicated certification helps to update medical knowledge is consistent with research exploring this aspect with emergency medicine physicians [7], family medicine physicians [4], rheumatologists [8], and CRNAs [14], whereby this benefit was also among the most frequently endorsed. In addition, related to learning occurring as a result of preparing for the exam, robust literature in cognitive psychology demonstrates that testing itself enhances learning by increasing information recall and retention [19, 20].

The second principal finding is that PA perspectives varied by age. Age 55 and older compared to less than 30 was among the strongest predictors of less favorable views, even after controlling for all other covariates. In the open-ended comments, PAs shared that recertification becomes more difficult over the course of their career, particularly when practicing in a very specialized discipline. A close examination of the literature on the association of age with perceptions of value of certification did not reveal a consistent pattern. For example, our finding contrasts with data from the National Board of Certification and Recertification for Nurse Anesthetists' (NBCRNA) report that showed CRNAs having more favorable perceptions of their credential with increasing age [14]. Many prior studies with physicians did not assess for potential differences in value of certification by age or a surrogate for age such as years in practice [7, 8, 10–12]; however, two studies found no significant differences [3, 21]. In the Peabody et al. study, there were no differences by age, but the authors did detect a relationship by years in practice [4]. Family medicine physicians practicing for 30 or more years had lower odds of reporting that they were motivated to continue their certification for extrinsic (e.g., required for hospital privileges/credentialing) rather than intrinsic (e.g., updating medical knowledge) reasons than those practicing up to ten years [4]. In another study with family medicine physicians, it was determined that older age at initial certification was associated with not attempting recertification [22]. Family medicine physicians who were initially certified when they were 40 or older had almost three-fold higher odds of not attempting recertification than those less than 30 [22]. Given these mixed findings, more research is needed to elucidate the relationship between age and perceptions of value of certification among different healthcare professions.

Aside from age being a significant predictor, we found that PAs from underrepresented in medicine (URiM) backgrounds had more favorable views regarding four of the eleven certification perceptions we assessed. For example, African American PAs, compared to white, were more likely to report that certification helps to update medical knowledge, monitor or improve patient care, advance professionally, and compete with other providers for clinical positions. Peabody and colleagues found differences in motivating factors for continuing participation in ABFM certification by race [4]. After controlling for other covariates, Asian and African American family physicians had lower odds of extrinsic motivation (required by employer, hospital privileges/credentialing, payer/insurance company) when compared to white. URiM medical practitioners are more likely to provide care in medically underserved communities and contribute to reducing health disparities [23]. URiM providers are also more likely to experience bias and discrimination in their workplace, including from colleagues and patients [24, 25]. It is critically important that PAs from URiM backgrounds are supported and derive benefits from certification. There is an ongoing need for increasing diversity in the PA profession [26], with the majority of PAs (over 80%) being white and 94% non-Hispanic/Latino [18]. Increasing health workforce diversity fosters innovation, improves patient access to care, satisfaction, and outcomes while reducing health disparities [27]. Strong and urgent efforts to diversify the racial and ethnic composition of the PA workforce are needed, particularly in light of the rapidly increasing diversity of the US population. Although favorable perceptions of certification are unlikely to directly increase diversity in the PA profession, it is encouraging that PAs from URiM backgrounds find certification valuable. Comments from a diverse group of PAs reflected that certification increases opportunities, legitimacy, respect, pride, and flexibility and helps to stay competitive and up-to-date. It may be that for some, certification helps demonstrate legitimacy, especially when experiencing racial/ethnic discrimination in the workplace. More in-depth qualitative research is needed to better understand perceptions of certification among PAs from URiM backgrounds.

Third, we found that PAs practicing in dermatology and psychiatry had less positive perceptions when compared to primary care. Reasons for the observed differences between specialties are speculative. The PANCE evaluates general medical knowledge, and the PANRE, along with the pilot, assess core medical knowledge. PAs working in psychiatry have reported needing a specialty-specific credential for credentialing and reimbursement purposes; therefore, the general certification may not seem as valuable to this group of PAs. In addition, it may be that dermatology and psychiatry are two of the more specialized disciplines, and PAs practicing in these specialties may perceive certification programs assessing a wide variety of diseases and disorders as less relevant to the specific circumstances of their practice. Hence, this may be why PAs practicing in primary care reported deriving more benefits. In addition, the general certification may not sufficiently address all of the credentialing needs for PAs working in these specialties. To help address credentialing needs for PA working in psychiatry, a Certificate of Added Qualifications (CAQ) was developed by NCCPA in 2011, and a CAQ in dermatology will be available in 2023. In our qualitative data, we found that many PAs in specialties and sub-specialties mentioned that being tested on core medical knowledge was not relevant to their current practice.

Our study has multiple limitations to consider. First is the potential for response bias. Although the response rate was high and PA participants were similar in demographics and practice characteristics to that of the overall national certified PA workforce, PAs who responded to the survey may have had stronger beliefs toward certification than those who chose not to participate. Another limitation potentially present in all survey research is social desirability bias, or the propensity to provide more socially acceptable responses rather than offering a frank opinion. We included a statement that the survey was confidential and results would be shared in aggregate to limit this bias. Nonetheless, some PAs may not have been comfortable responding to questions about how they view certification in a survey administered by NCCPA. A third limitation is that we assessed PAs participating in the pilot program on the global value of certification rather than including PAs participating in all certification exam programs (i.e., PANCE, PANRE, Pilot). Remarks to the open-ended question suggest that PAs may have different perspectives regarding each of these exams that are used for initial certification or for maintaining certification. Further, PAs participating in the pilot may have different views from PAs taking traditional PANRE.

## Conclusions

Overall, the picture that emerged is that PAs value certification, but perceptions varied by demographics and specialties. PAs who were younger, from URiM backgrounds, and practicing in primary care specialties had among the most favorable perspectives. However, this study's more important takeaways may be the reasons PAs value certification and the potential impact on patient care. A large majority of PAs indicated that certification helps them stay current with medical knowledge and provides objective evidence of competencies. In addition to these reasons, PAs from URiM backgrounds noted that certification also helped them advance professionally and compete with other providers for clinical positions. Continued feedback monitoring will play a critical role in ensuring PAs continue to derive benefits from certification. Measuring PA perceptions of the value of certification is essential to understand what works and how to continue positive trends that may ultimately support improved patient care.

## Data Availability

The datasets generated and analyzed during the current study will not be shared due to confidentiality of individualized data, but deidentified data can be available if requested from the authors.

## Abbreviations

PA: Physician assistant/associate
NCCPA: National Commission on Certification of Physician Assistants
URiM: Underrepresented in Medicine
MOC: Maintenance of certification
PANCE: Physician Assistant National Certifying Exam
PANRE: Physician Assistant National Recertifying Exam
PANRE-LA: Physician Assistant National Recertifying Exam-Longitudinal Assessment
CRNA: Certified Registered Nurse Anesthetist
NP: Nurse practitioner
CAQ: Certificate of Added Qualifications
